# Does Progesterone Affect Perioperative Blood Loss during Posterior Spinal Fusion Surgeries in Female Patients with Adolescent Idiopathic Scoliosis? A Retrospective Study

**DOI:** 10.1111/os.13730

**Published:** 2023-04-18

**Authors:** Yang Jiao, Haining Tan, Zhen Wang, Junduo Zhao, Jianxiong Shen

**Affiliations:** ^1^ Department of Orthopedics Peking Union Medical College Hospital, Peking Union, Medical College, Chinese Academy of Medical Science Beijing China

**Keywords:** Adolescent idiopathic scoliosis, Perioperative blood loss, Posterior spinal fusion surgery, Progesterone

## Abstract

**Objective:**

Menstruation is considered a contraindication for elective surgery for higher operative blood loss. Progesterone is often used to postpone menstruation to avoid surgery during menstruation. This study aimed to explore whether using progesterone to postpone menstruation affects perioperative blood loss and complications in female patients with adolescent idiopathic scoliosis (AIS) who underwent posterior spinal fusion (PSF) surgery.

**Methods:**

A retrospective study was performed for female patients diagnosed with AIS who underwent PSF surgery between March 2013 and January 2021. Patients scheduled to undergo PSF surgery from 2 days before menstruation to 3 days after menstruation were treated with progesterone preoperatively. The patients were divided into two groups according to progesterone use (progesterone injection group; control group). Demographic and surgical data including intraoperative blood loss (IBL), normalized blood loss (NBL), total blood loss (TBL), transfusion rate, perioperative complications, postoperative drainage time, postoperative hospital stay, and preoperative coagulation function data were collected.

**Results:**

A total of 206 patients were included in the study. Among them, the progesterone injection group included 41 patients, with an average age of 14.8 years. While the control group included 165 patients, with an average age of 14.9 years. The two groups were matched for age, height, weight, operation time, Risser sign, correction rate, mean curve Cobb angle, bending Cobb angle, number of internal fixations, and number of fused levels (all *P* > 0.05). Regarding coagulation function, no significant differences were found in thrombin time, activated partial thromboplastin time, fibrinogen, prothrombin time, and platelet count between the two groups (all *P* > 0.05). IBL, NBL, and TBL were higher in progesterone injection group; however, the difference was nonsignificant (all *P* > 0.05). Transfusion rate, perioperative complications, postoperative drainage time, and postoperative hospital stay were not statistically different between groups (all *P* > 0.05).

**Conclusion:**

Intramuscular injection of progesterone to avoid menstruation during PSF surgery did not affect perioperative blood loss and complications in AIS patients. It may be a safe method for AIS patients to avoid menstrual problems affecting the operation time and receive PSF surgery as scheduled.

## Graphical abstract

The flowchart of enrolled AIS patients. Abbreviations: AIS: adolescent idiopathic scoliosis.

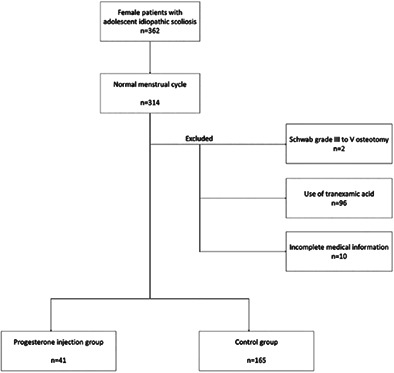

## Introduction

An estimated 1%–4% of adolescents in early puberty suffer from adolescent idiopathic scoliosis (AIS), which is more common in young women.^1^ Female/male ratios can be as high as 10:1 for patients with Cobb angles greater than 30°.^2^ Most AIS patients who require surgical intervention are women.

For patients with AIS, posterior spinal fusion surgery (PSF) is usually required to correct spinal deformities and reconstruct spinal stability.^1^ Due to the large surgical wound, long operation time and other factors, the risk of massive blood loss during PSF surgery is high.^3^, ^4^, ^5^ Massive blood loss can result in hemodynamic complications and transfusion‐related complications.^5^, ^6^, ^7^ Studies have shown that patients' developmental and surgical factors are risk factors for blood loss during PSF.^8^, ^9^, ^10^, ^11^ Furthermore, many surgeons believe that menstruation is a contraindication for surgery because the functional impairment of the coagulation system may increase surgical blood loss, and the accompanying pain may delay the diagnosis of perioperative complications.^12^, ^13^, ^14^

Progesterone, as one of the main hormones that affect the menstrual cycle, is often used to postpone menstruation before surgery to avoid canceling scheduled PSF surgery for female AIS patients. In addition to regulating the menstrual cycle, progesterone has been shown to affect blood coagulation.^15^, ^16^ The use of progesterone and synthetic analogs, progestogens, in the treatment of abnormal uterine bleeding is well established.^17^ Previous researches on progesterone have mainly focused on its therapeutic effects on hormone‐dependent organs such as hemostasis, maintaining pregnancy, and contraception, as well as its association with thrombotic risk.^15^, ^16^, ^17^ However, there are no published studies investigating whether using progesterone to postpone menstruation affects perioperative blood loss and complications in PSF surgery.

The purpose of this study was to investigate whether using progesterone to postpone menstruation affects: (i) perioperative blood loss; and (ii) the incidence of perioperative complications in female AIS patients who underwent PSF surgery.

## Methods

This retrospective study was approved by the Peking Union Medical College Hospital's Ethical Committee (IRB/IEC no. S‐K1669). The inclusion criteria were: (i) female AIS patients; (ii) patients who underwent PSF surgery at a single institution between March 2013 and January 2021; and (iii) operation was performed by the same senior spinal surgeon. The exclusion criteria were: (i) irregular menstruation or before menarche; (ii) underwent anterior or revision surgery; (iii) underwent Schwab grade III‐VI osteotomy; (iv) used tranexamic acid (TXA); (v) with abnormal blood coagulation function; and (vi) with abnormal indicators of renal or liver insufficiency.

AIS was diagnosed based on Weinstein *et al*.'s description.^1^ Patients enrolled in this study were divided into two groups according to the use of progesterone: progesterone injection group, patients who received progesterone injection preoperatively; and control group, patients who underwent PSF surgery during the non‐menstrual period and did not receive progesterone injection preoperatively.

Preoperative data, including age, weight, height, Risser sign, Lenke curve classification, Cobb angle of major curve, bending Cobb angle, albumin, hemoglobin, hematocrit levels, platelet count, activated partial thromboplastin time (APTT), thrombin time, prothrombin time, and fibrinogen, were retrospectively collected from each case. Based on the formula by Brecher *et al*.,^18^ $\left\{\mathrm{TBV}\;\left(\mathrm{mL}\right)=\text{weight}\;\left(\mathrm{kg}\right)\times 65\right\}$, preoperative total blood volume (TBV) was calculated for each case. Menstruation‐related data, including duration of the menstrual cycle, duration of menstruation, and date of last menstruation, were obtained from medical records.

### The Use of Progesterone

Patients received progesterone treatment if their scheduled operation date was within 2 days before menstruation and 3 days after menstruation to postpone the menstruation and perform PSF surgery on schedule. We selected a relatively low‐dose progesterone administration protocol based on the pharmacokinetics of the drug and previous studies. Progesterone is usually administered to treat premenstrual syndrome at 200–400 mg daily for 2 weeks by vaginal or oral administration.^19^ For menstrual disorders, progesterone is often used to postpone or advance menstruation.^17^ The general dosage was oral progesterone 100–400 mg daily, or intramuscular injection progesterone 20 mg daily for 5–6 days.^17^, ^20^ Furthermore, 200 mg progesterone is usually administered vaginally daily or 250 mg weekly through intramuscular injection for weeks to prevent preterm birth.^21^ In our study, patients in the progesterone injection group received a dose of 20 mg progesterone from 3 days before surgery to the operation day. The drug was administered through intramuscular injection once a day. In the control group, no patient received progesterone or other sex hormones (estrogen, progestogen, or others).

### Anesthetic and Surgical Procedures

The operation was performed under general intravenous anesthesia induced and maintained using standard agents. A cell‐saver system was used for patients in need. Intraoperative blood transfusion was initiated when intraoperative hemoglobin (Hgb) was lower than 7.0 g/dL, mean intraoperative arterial blood pressure (ABP) was less than 50 mmHg, or urine output was significantly decreased. Patients received blood transfusion had their Hgb, ABP, and urine volume changes re‐examined after transfusion.

The fusion levels were selected using the same criteria in both groups. The spinal cord monitor was applied in all cases during surgery. Routine posterior exposure and Schwab grade I or II osteotomies were used to resect bilateral facet joints within the fusion range. Pedicle screw constructs were used in all PSF surgeries. The pedicle screws were polyaxial in shape. The correction was performed using rod rotation, vertebral derotation, and the concave side distraction/convex side compression techniques. After correction, the lamina in the range of fusion was decorticated, and morselized allogeneic bone combined with autogenous bone was used for bone graft fusion. A deep subfascial drain was inserted before closure. Iliac crest bone grafts were not used in this study. Time between the initial incision and wound closure was defined as the operation time.

### Postoperative Treatment

Patients in both groups underwent the same postoperative protocol. The wound drain was removed when the drainage volume was below 60 mL/day. The postoperative drainage time is the time between surgery and drainage tube removal. Postoperative transfusion was performed when the Hgb level was <7.0 g/dL, or the patient had clinical symptoms, such as palpitations caused by anemia.

### Outcome Measurements

PSF surgery‐related data, including operation time, number of fused vertebrae and pedicle screws, amount of intraoperative crystalloid, ABP, transfusion volume, and blood volume collected by the cell‐saver, were collected. By adding the blood volumes collected from the suction and cell‐saver systems and weighing the surgical sponges, intraoperative blood loss (IBL) was calculated. The total perioperative estimated blood loss (TBL) was calculated by adding IBL to the volume of blood obtained from the drains postoperatively. Normalized blood loss (NBL) was recorded as $\mathrm{TBL}/\left(\text{weight}\times \text{number of fusion levels}\right)$. Data on postoperative day 1 hematocrit (Hct), Hgb, drainage time, hospital stay, and perioperative complications were obtained for each case. The postoperative correction rates for scoliosis were calculated for each case.

### Statistical Analysis

Quantitative data are expressed as the mean ± standard deviation. The student's *t*‐test was used for continuous variables. Chi‐square tests were used to analyze categorical data. The Kruskal–Wallis test was used to determine the number of fusion levels and internal fixations. Statistical significance was set at *P* < 0.05. SPSS (version 24.0; IBM, Armonk, NY, USA) was used for statistical analyses.

## Results

### General Results

**Fig. 1:**
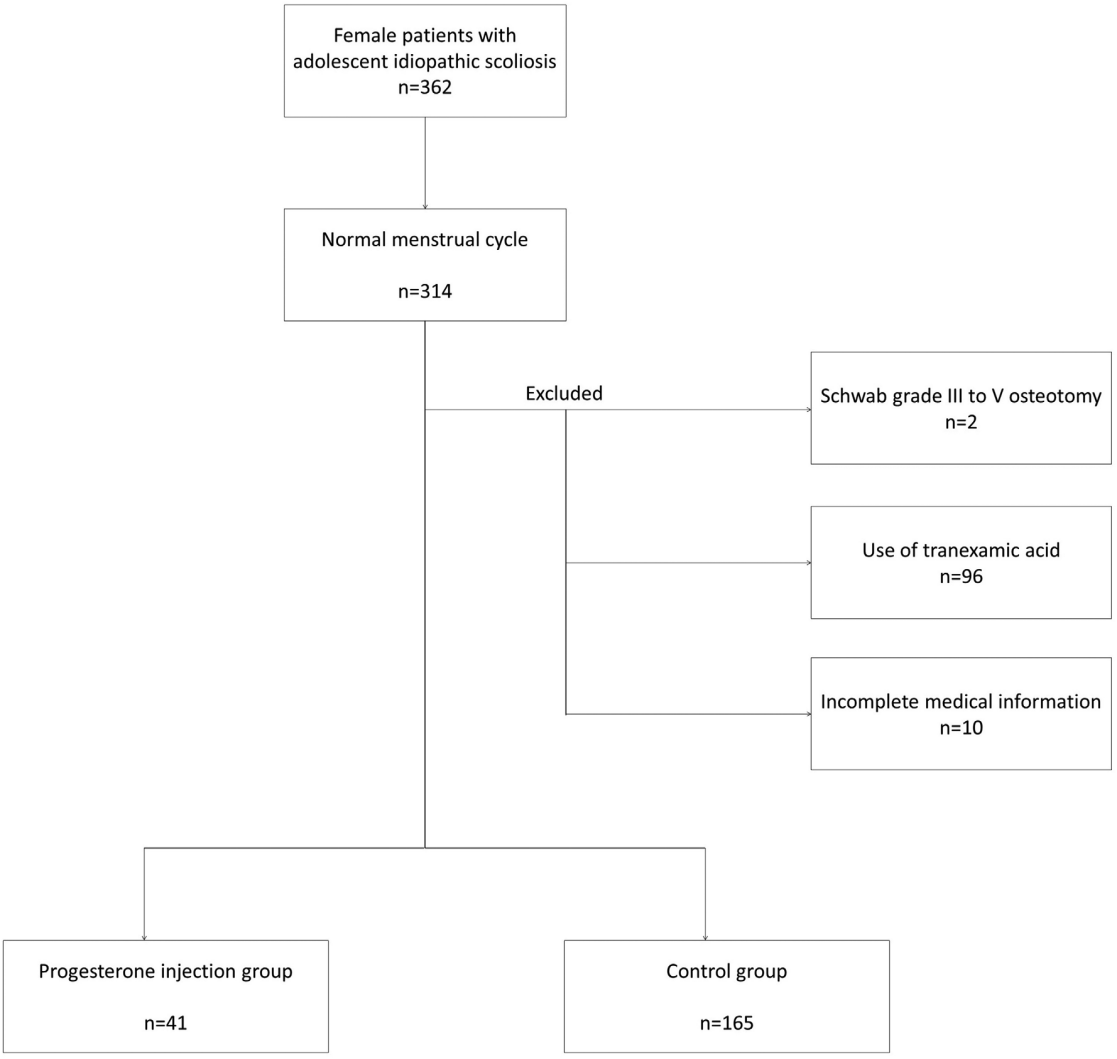
The flowchart of enrolled patients.

We screened 362 female AIS patients who underwent PSF surgery. Among them, 156 were excluded because of Schwab grade III‐VI osteotomy (two), TXA or other hemostatic drugs (96), menstrual cycle issues (48: before menarche, or irregular menstrual cycle), and incomplete medical information (10). Study participants included the 206 remaining patients. Among them, 41 were included in progesterone injection group and 165 in control group (Fig. 1). No statistically significant differences were found in the demographic and radiographic data (age, height, weight, total blood volume, Risser sign, Lenke classification, Cobb angle, and bending Cobb angle between the two groups, Table 1). Preoperative coagulation function variables, including platelet count, fibrinogen level, prothrombin time, thrombin time, and APTT, are presented in Table 2. Coagulation functions were not significantly different between the two groups.

**TABLE 1:** Demographic and radiographic data

	Progesterone injection group	Control group	t	*p*
	*n* = 41	*n* = 165		
Age (year)	14.8 ± 2.2	14.9 ± 1.8	−0.45	0.64
Height (cm)	161.2 ± 6.4	160.4 ± 6.7	0.209	0.27
Weight (kg)	47.8 ± 7.1	48.6 ± 7.4	1.354	0.43
Total blood volume (mL)	3107 ± 853	3159 ± 1004	0.945	0.40
Risser sign	3.6 ± 1.1	3.7 ± 0.9		0.79
Major Cobb angle (°)	53.9 ± 15.9	52.8 ± 15.2	−1.825	0.16
Bending Cobb angle (°)	29.7 ± 21.2	29.2 ± 16.1	−1.817	0.28
Lenke classification				
1	18	80		0.68
2	9	37		0.53
3	3	11		0.66
4	1	4		0.43
5	6	19		0.34
6	4	14		0.26

*Note*: Statistically significant (*P* < 0.05).

**TABLE 2:** Preoperative coagulation function variables between progesterone injection group and control group

	Progesterone injection group	Control group	t	*p*
	*n* = 41	*n* = 165		
Platelet (×10^9^/L)	221.8 ± 57.3	243.7 ± 71.9	1.893	0.40
Prothrombin time (s)	12.6 ± 0.8	12.2 ± 0.5	−0.969	0.91
Thrombin time (s)	17.5 ± 0.9	17.9 ± 0.8	1.492	0.56
APTT (s)	31.5 ± 3.2	32.7 ± 3.9	1.41	0.74
Fibrinogen (g/L)	2.4 ± 0.4	2.7 ± 0.5	−1.897	0.32

Abbreviation: APTT, activated partial thromboplastin time.

*Note*: Statistically significant (*P* < 0.05).

### Surgery‐related Results

The surgery‐related variables between the two groups showed no significant differences in IBL, TBL, NBL, operation time, mean intraoperative arterial pressure, intraoperative volume of crystalloid, cell‐savor usage (%), cell‐saver transfusion volume, correction rate, transfusion rate, drainage time and postoperative hospital stay (Table 3). The Kruskal–Wallis test showed no difference in the number of fusion levels (*P* = 0.19) and internal fixations (*P* = 0.26). In terms of correction rate, no significant difference was found between the progesterone injection group (65.9% ± 12.6%) and the control group (66.3% ± 10.9%). The mean operative time was 202 ± 60 min in the progesterone injection group and 209 ± 56 min in the control group (*P* = 0.17). Six patients in the progesterone injection group (14.6%) and 12 in the control group (7.3%) required allogeneic blood transfusions during or after surgery; however, the difference was not statistically significant.

**TABLE 3:** Surgery‐related variables between progesterone injection group and control group

	Progesterone injection group	Control group	*t*	*p*
	*n* = 41	*n* = 165		
Intraoperative blood loss (IBL), mL	715.6 ± 348.3	649.5 ± 311.5	−1.447	0.25
Normalized blood loss (NBL)				
{TBL (mL) /Weight (kg) × No of fusion level}	1.5 ± 0.9	1.4 ± 1.2	−2.084	0.12
Total blood loss (TBL), mL	958 ± 329	894 ± 384	−0.63	0.76
Operation time (min)	202.9 ± 60.2	209.6 ± 56.1	0.116	0.17
Mean ABP (mm Hg)	71.5 ± 5.2	72.4 ± 6.6	1.677	0.12
Crystalloid (mL)	1420 ± 615	1490 ± 907	0.875	0.89
Cell‐saver (% used)	90.2%	93.3%		0.72
Cell‐saver transfusion (mL)	225 ± 116	210 ± 152	0.574	0.27
Number of pedicle screw	15.0 ± 2.8	14.8 ± 3.0		0.52
Number of fusion level	9.7 ± 2.4	9.6 ± 2.6		0.19
Postoperative Cobb angle (°)	18.4 ± 7.8	17.8 ± 9.8	−1.253	0.26
Correction rate (%)	65.9 ± 12.6	66.3 ± 10.9	1.116	0.89
Postoperative drainage time, day	2.61 ± 0.8	2.59 ± 0.7	−0.654	0.44
Postoperative hospital stay, day	6.0 ± 1.2	5.8 ± 1.2	−0.372	0.35
Preoperative hemoglobin, g/L	13.5 ± 1.9	13.3 ± 1.1	0.517	0.37
Postoperative hemoglobin, g/L	9.6 ± 2.0	10.1 ± 1.3	0.734	0.68
Transfusion rate (%)	14.6	7.3		0.46

Abbreviations: ABP, arterial blood pressure; No, number.

*Note*: Statistically significant (*P* < 0.05).

As for the perioperative blood loss. The IBL, TBL, and NBL of the progesterone injection group were 715 ± 348, 958 ± 329, and 1.5 ± 0.9 mL/kg × fusion level, respectively. While the IBL, TBL, and NBL of the control group were 649 ± 311, 894 ± 384, and 1.4 ± 1.2 mL/kg × fusion level, respectively. The IBL, TBL, and NBL of the progesterone injection group were higher than those of the control group, however, these differences were not statistically significant (all *P* > 0.05).

### Complication

One patient in control group developed delayed wound infection 12 months after surgery, and internal fixations were later removed. Another patient in control group developed superior mesenteric artery syndrome 6 days after operation, which was later recovered after 5 weeks of diet adjustment treatment. None of the patients in progesterone injection group sustained any major perioperative complications. However, five patients experienced nausea a few hours after progesterone injection, which could be relieved after symptomatic treatment. No other adverse effects associated with progesterone use, such as headaches or diarrhea, were noted. None of the patients suffered from deep venous thrombosis or liver complications.

## Discussion

The result of our study indicated that preoperative injection of progesterone to avoid menstruation during PSF surgery did not affect perioperative blood loss or complications in AIS patients.

### Menstruation and PSF Surgery

Surgery during menstruation is a common concern for surgeons because of the malfunctioning coagulation system during menstruation.^12^ Findikcioglu *et al*.^14^ reported that operative blood loss is higher during the periovulatory phase of rhinoplasty surgery. As reported by Sariguney *et al*.,^13^ perioperative blood loss in breast reduction surgery is greater during the perimenstrual phase than during the periovulatory period. However, some studies have shown contradictory results. A study by Lin *et al*.^22^ found that menstruation had no significant effect on operative bleeding in vitreoretinal surgery. It was found by Das *et al*. that women undergoing open heart surgery during menstruation did not experience any increase in either surgical or menstrual blood loss.^23^

There are few studies on menstruation and perioperative blood loss in PSF surgery. A study by Li *et al*.^24^ analyzed 161 cases of AIS by grouping them into four categories: premenstrual (24–30 days), follicular (6–11 days), ovulatory (12–17 days), and luteal (18–23 days). They found that female AIS patients who underwent PSF surgery during the premenstrual phase of the menstrual cycle suffered more IBL. In the study, menstruation was considered a contraindication for PSF surgery; thus, they avoided performing it on patients during menstruation due to concern about increased perioperative blood loss. However, Chiu *et al*.^25^ compared AIS patients who underwent PSF during the menstrual period with those who underwent PSF during the non‐menstrual period and found no significant difference in IBL. In our institution, PSF surgery is usually not performed during menstruation for the following reasons. First, PSF surgery is characterized by a large IBL; since during menstruation patients lose a significant amount of blood, this increases their odds of needing blood transfusion. Second, many AIS patients are not local residents, and when the operation date conflicts with their menstrual date, if they wait until the end of their menstruation to undergo PSF surgery, this usually translates into financial and emotional complications for both the patients and their guardians. Third, pain and fatigue associated with menstruation will delay the diagnosis of complications and affect postoperative rehabilitation exercise. As progesterone withdrawal triggers menstruation, we used progesterone to postpone menstruation and staggered the PSF surgery and menstruation. Menstruation usually occurs on the third or fourth day after the last progesterone injection, and rehabilitation exercise is usually restored in patients with AIS who underwent PSF surgery.

### Progesterone Did Not Increase the Incidence of Complications

While progesterone can effectively postpone menstruation, some studies have suggested it may increase thrombotic risk. Previous studies have shown that oral contraceptives (including estrogen and progesterone) can increase plasma concentrations of fibrinogen, factor VIIa, factor VIII, free protein S, and APTT‐based activated protein C sensitivity ratio, which might increase the risk of thrombotic situations.^15^, ^26^ A higher‐quality study also suggested that estrogen plus progestogen users have a higher risk of thrombosis than estrogen users alone.^27^ As a hormone replacement therapy, Canonico *et al*. found that progesterone alone increased the risk of venous thrombosis in postmenopausal women.^28^ However, the role of progesterone in venous thromboembolism is controversial. In premenopausal women, Vasilakis *et al*. found no increase in thrombosis risk when progesterone was used alone for contraception.^29^ Through network meta‐analysis, Stegeman *et al*. found that the difference in the incidence of venous thrombosis outcomes between women using oral contraceptives and those not using them was only 1.4 per 10,000.^16^ The discrepancy between progesterone and thrombosis results may be due to the different levels of physiological sex hormones in the study population. In addition, the form of administration, dosage, frequency, and duration of progesterone treatment differed. In our study, all patients who received progesterone treatment had their menstruation successfully postponed. However, we did not find that progesterone injection increased the risk of thrombus‐related events. Our results were basically consistent with those of Vasilakis *et al*. ^29^ and Stegeman *et al*.^16^ In addition, there was no difference in complications such as infection, prolonged hospital stay, and delayed wound healing between the two groups. It is worth noting that there were no major perioperative complications in the progesterone injection group. This may be due to the short‐term (4 days) and relatively small doses (20 mg/day) of progesterone employed, compared to the longer courses and higher dosages of progesterone used for other indications.

### Perioperative Blood Loss Was Not Significantly Different between the Two Groups

In our study, we minimized the confounding factors associated with perioperative blood loss. All patients underwent surgery performed by the same senior spinal surgeon at our hospital. Perioperative treatment was performed following the same protocol. Patients who underwent Schwab grade III‐VI osteotomy and those who received TXA treatment were excluded from this study. This is because high‐grade osteotomy has been proven to be a risk factor for blood loss in PSF surgery.^30^ TXA has also been proven to reduce blood loss and blood transfusion rates in PSF surgery for AIS patients.^31^ All demographic and surgical factors were statistically analyzed to be matched between the two groups. Eventually, the IBL was slightly (numerically) higher in the progesterone injection group than in the control group (715 ± 348 vs. 649 ± 311 mL); however, the difference was not statistically significant. Furthermore, there was no significant difference in TBL between the two groups. To avoid the influence of weight and the number of fusion levels, NBL was calculated; however, there was still no significant difference even after considering these variables. Our results were similar to those of Chiu *et al*.,^25^ but their study group was AIS patients underwent PSF surgery during menstruation. Allogeneic blood transfusion rates were higher in progesterone injection group (14.6%) than in control group (7.3%); however, this difference was not statistically significant either. Therefore, we postulate that using progesterone to postpone menstruation does not affect perioperative blood loss in AIS patients undergoing PSF surgery.

### Limitations and Strengths of this Study

This study has several limitations. First, it was limited by the inherent limitations of a retrospective study. The use of progesterone during PSF surgery needs to be evaluated in a prospective randomized study in order to make cause‐and‐effect inferences. Multicenter studies can be conducted to further explore the effect of progesterone on PSF surgery. Second, no hormonal studies were performed. Since the menstrual cycle is regulated primarily by the four hormones estrogen, luteinizing hormone, progesterone, and follicle‐stimulating hormone, further studies are needed to explore which hormone is more suitable for postponing menstruation in PSF surgery and its appropriate dose and administration schedule. The strength of our study was that it was the first to investigate the effect of progesterone on perioperative blood loss and complications in PSF surgery.

### Conclusion

This retrospective study found that intramuscular injection of low‐dose progesterone to avoid menstruation during PSF surgery did not increase IBL, NBL, TBL, and the incidence of perioperative complications in AIS patients. Therefore, this method seems safe and can continue to be used in female patients with AIS.

## Author Contributions

YJ collected data from medical records. HT reviewed the radiographs. JZ performed the statistical analyses. YJ wrote the manuscript. ZW provided intellectual support. JS finalized the manuscript, and was responsible for this. All authors reviewed the manuscript. Final approval of the manuscript has been obtained from all authors.

## Ethics Approval and Consent to Participate

All patients/participants and their legal guardians provided written informed consent. Peking Union Medical College Hospital's Ethical Committee approved the study. Helsinki Declaration principles were followed in the study protocol.

## Data Availability

On reasonable request, the corresponding author will provide the dataset supporting the conclusions of this study. An administrative permission was obtained from Peking Union Medical College Hospital for access to medical records.
